# Trophic structure and energy flow in a shallow-water hydrothermal vent: Insights from a stable isotope approach

**DOI:** 10.1371/journal.pone.0204753

**Published:** 2018-10-17

**Authors:** Ni-Na Chang, Li-Hung Lin, Tzu-Hsuan Tu, Ming-Shiou Jeng, Yoshito Chikaraishi, Pei-Ling Wang

**Affiliations:** 1 Department of Geosciences, National Taiwan University, Taipei, Taiwan, ROC; 2 Institute of Oceanography, National Taiwan University, Taipei, Taiwan, ROC; 3 Biodiversity Research Center, Academia Sinica, Taipei, Taiwan, ROC; 4 Institute of Low Temperature Science, Hokkaido University, Kita-ku, Sapporo, Japan; 5 Institute of Biogeosciences, Japan Agency for Marine-Earth Science and Technology, Yokosuka, Japan; Centro de Investigacion Cientifica y de Educacion Superior de Ensenada Division de Fisica Aplicada, MEXICO

## Abstract

Shallow-water hydrothermal vent ecosystems are distinct from the deep-sea counterparts, because they are in receipt of sustenance from both chemosynthetic and photosynthetic production and have a lack of symbiosis. The trophic linkage and energy flow in these ecosystems, however remain elusive, which allows us poor understanding of the whole spectrum of biological components distributed across such environmental gradients. In this study, a thorough isotopic survey was conducted on various biological specimens and suspended particulates collected along four transects across the venting features of a shallow-water hydrothermal field off Kueishan Island, Taiwan. The isotope data combined with a Bayesian-based mixing model indicate that the vent-associated particulate organic matter (vent POM), as primary contribution of chemoautotrophic populations, has a high δ^13^C value (−18.2 ± 1.1‰) and a low δ^15^N value (−1.7 ± 0.4‰). Zooplankton and epibenthic crustaceans, as the fundamental consumers, exhibit δ^13^C and δ^15^N values ranging from −21.3 to −19.8‰ and +5.1 to +7.5‰, respectively, and can utilize the vent POM for 38–53% of their diets. The vent-obligate crab *Xenograpsus testudinatus* shows a large variation in δ^13^C (from −18.8 to −13.9‰) and δ^15^N values (from 1.1 to 9.8‰), although an omnivorous trophic level (2.5) is identified for it using δ^15^N values of amino acids, and it can utilize the vent POM for 6–87% of its diet. The consistently low (< 10.0‰) and overlapping δ^15^N values for most of the analyzed macroinvertebrates suggest extensive ingestion of chemosynthetic production complementing the photosynthetic production, a weak prey–predator relationship and low trophic complexity possibly imposed by the extreme environmental contexts of shallow-water hydrothermal ecosystems.

## Introduction

Studies of hydrothermal vent ecosystems distributed along mid-ocean ridges and back-arc spreading centers have greatly expanded our knowledge regarding the energy sources of marine communities. Unlike the vast majority of marine ecosystems sustained by photosynthetic primary production, benthic ecosystems associated with deep-sea hydrothermal vents are primarily driven by chemoautotrophic production that harvests metabolic energy from the oxidation of abundant reducing compounds (e.g., CH_4_, H_2_S, and NH_4_^+^) released from venting features [1,2]. The discharge of hot, reducing fluids into the cold, oxidized deep sea facilitates the generation of strong redox and temperature gradients, both of which favor the colonization of microbial communities possessing various physiological characteristics. These chemoautotrophic microbes efficiently sustain vent communities *via* bacteria–invertebrate symbioses or heterotrophic consumption, rendering deep-sea vent ecosystems analogous to oases in the desert [3]. Determining the energy flow would, therefore, provide an important basis to quantify the potential export of geothermal energy and chemical fluxes from vent ecosystems to the open ocean [4,5].

Distinct from their deep-sea counterparts, shallow-water hydrothermal vents occur in coastal euphotic zones. Therefore, photosynthesis could contribute to primary production at quantities comparable with or even exceeding chemosynthesis. Previous studies have demonstrated that 15–60% of the primary production in shallow-water hydrothermal fields could be accounted for by photosynthesis (Kraternaya Bight, [6]). Besides, shallow-water vent ecosystems are generally deprived of endosymbiont-containing and vent-obligate species commonly observed in deep-sea vents [2,4,7,8], and instead are composed of a reduced subset of the surrounding species [9,10]. The inherent differences in the functional nature of primary producers and species composition may potentially distinguish these two counterparts in various aspects, such as food utilization and trophic structures [6,11]. However, most of the studies of shallow-water vent ecosystems have not progressed much beyond the taxonomic description of species [12,13] and surveys of the community structure [14–16]. The respective contributions of photosynthetic and chemoautotrophic primary production to benthic and even pelagic communities are poorly understood and a quantitative picture of energy transfer in such ecosystems is required.

The ecosystem near Kueishan Island (aka Kueishan Tao; KST) off northeastern Taiwan provides a model field for studying energy flow in shallow-water hydrothermal vents. It is unique and rare in that the vent-obligate crab, *Xenograpsus testudinatus* (Crustacea: Brachyura), dominates over other benthic community members [17]. In addition, non-obligate macroinvertebrates common in intertidal and coastal ecosystems constitute a significant fraction of the ecosystem [16]. The benthic fauna possesses various affinities for hydrothermal activities and drastic dietary preferences for different types of food products, thereby potentially forming an interwoven trophic structure. Nevertheless, how chemosynthetic and photosynthetic production is transferred between different compartments distributed at different horizontal and vertical scales is not well constrained.

This study aims to investigate a thorough spectrum of food source utilization, trophic structure, and energy flow in the KST ecosystem. Particulate organic matter (POM), fauna, and macroalgae were collected across the venting features and analyzed for their bulk carbon and nitrogen isotopic compositions to quantitatively distinguish the respective contributions of chemosynthetic and photosynthetic production [18]. To better understand the energy utilization of the dominant species *X*. *testudinatus*, which is thought to occupy the highest trophic level in the KST hydrothermal ecosystem [19], the nitrogen isotopic compositions of amino acids were also analyzed to estimate the trophic position of these vent-obligate crabs without prior determination of the δ^15^N values of the primary producers. This isotopic survey identified potential food sources and consumers across both vertical and horizontal scales and established a general model of energy flow and benthic−pelagic coupling in shallow-water vent ecosystems.

## Materials and methods

### Study sites and field sampling

**Fig 1:**
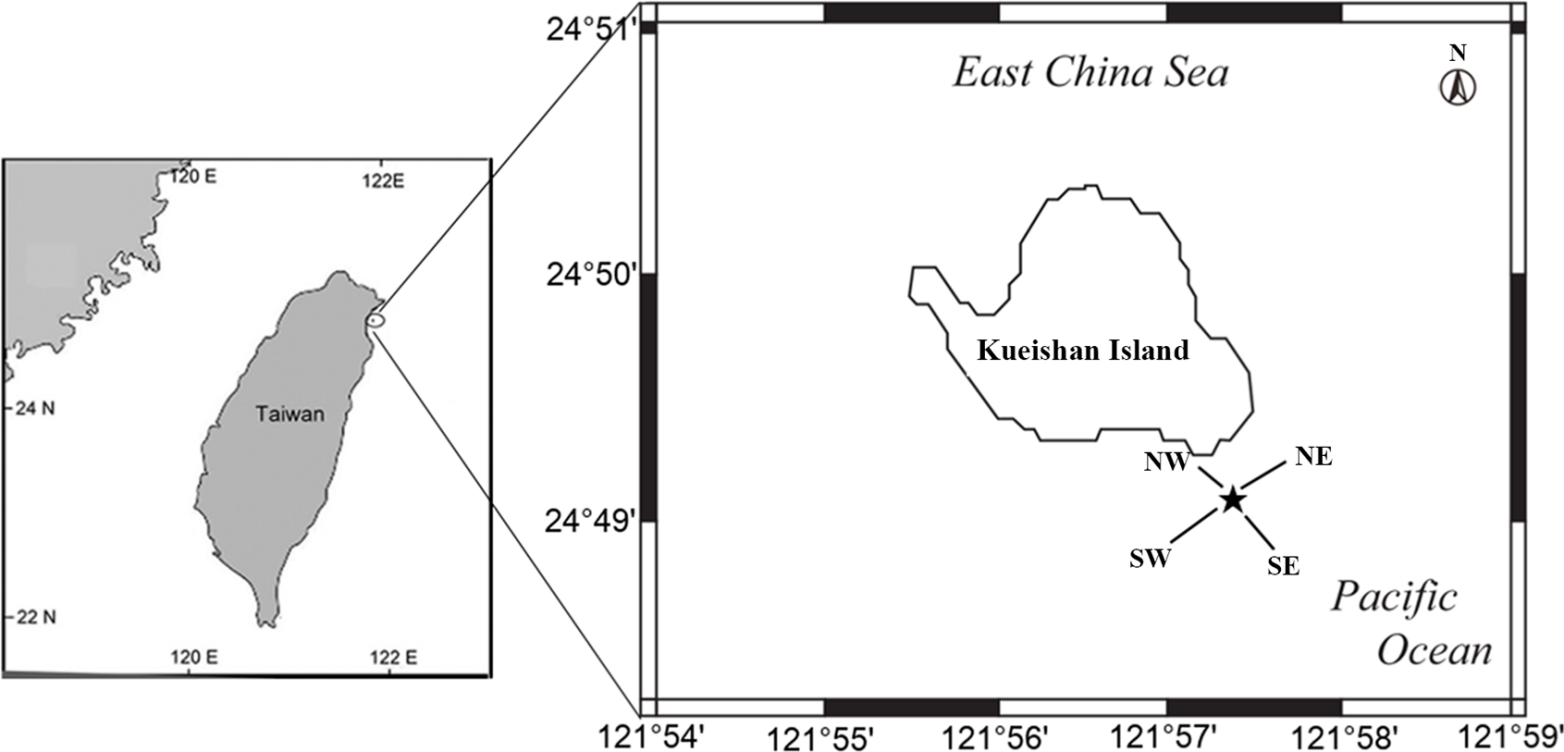
Sampling location of the Kueishan Island hydrothermal vent field. Star symbol indicates the center of the sampled hydrothermal vent. Solid lines represent four sampling transects (NE, SW, NW, and SE).

KST is located *ca*. 15 km off the northeastern coast of Taiwan and aligned with the southwestern extension of the Okinawa Trough. It is composed of andesitic rocks formed during the subduction of the Philippine Sea plate underneath the Eurasian continent and represents the most westward extension of volcanic activity within the Okinawa Trough [20] (Fig 1). The hydrothermal vent field with numerous venting features is located off the island to the east at a water depth of less than 30 m. The sites of venting features vary temporally, depending on whether the fracture channel is exposed at the seafloor. The emitted fluids are CO_2_-rich, extremely acidic (the lowest recorded pH in the world of 1.52 and 2.77 on average [20]) and hot (temperatures between 48°C and 116°C [17]). Helium isotope compositions (6 times the atmospheric ratio) indicate that these gases are magmatic in origin [21]. An extremely high density (> 360 ind/m^2^) of vent-obligate crabs, e.g., *X*. *testudinatus*, have been found to inhabit areas near the chimneys and rock crevices where gas bubbles emerge [17].

No specific permission was required for the sampling as the location is not private-owned or protected under any regulation, and this study did not involve endangered or protected species. One of the vents (N24° 50.054', E121° 57.714'; water depth of *ca*. 10 m) with continuous discharge of whitish fluids and sulfur deposited at the venting peripheral was chosen as the center for our sampling campaign conducted in August and September of 2015. Sampling was conducted along four transects (100 m in length with the exception of the shoreward NW transect) extending from the vent center to the peripheral region (shallower than 25 m) in order to collect samples with sufficient biological diversity and to uncover the effects of venting on trophic structure. The horizontal intervals for sampling benthos and seawater were 10 m and 20–50 m, respectively. The vent-influenced area is usually milky blue in color and its range exhibits great spatiotemporal variation depending on the tides and currents.

Various samples, including suspended POM (presumably planktonic microorganisms and algae), zooplankton, benthic fauna, and macroalgae were retrieved from different compartments of the ecosystem to provide a full spectrum of biological diversity for trophic reconstruction. Seawater (4 to 8 L) was collected from the sea bottom by scuba divers and from the sea surface by towing a bucket at each sampling site. Venting fluids (*ca*. 4 L) were collected from the venting orifice using a titanium pipe connected with a Teflon pipe and a pre-vacuumed sterile bottle. An extra venting fluid sample was collected in a close but different vent in October 2016 to verify the composition of vent fluids. The collected fluids were filtered using pre-combusted (500°C, 8 h) glass fiber filters (pore size of 0.7 μm) to collect POM for isotopic analysis. Zooplankton was collected by obliquely towing a 200-μm mesh plankton net at the sea surface in both vent-influenced and non-vent locations. The sampled zooplankton were then separated into five size categories (> 2000, 1000–2000, 500–1000, 363–500, and 200–363 μm) for further isotopic analyses using stainless steel sieves. For small epibenthic crustaceans, a light trap equipped with a 5-L plastic bottle and a light source was deployed overnight on the seabed of both venting and non-vent areas. Benthic macrofauna, including the predominant vent crab (*X*. *testudinatus*) and sea anemones (*Anthopleura* sp.) species and other coexisting species including corals (*Tubastraea* sp.) and sea snails (*Anachis misera* and *Ergalatax contractus*), were collected along the transects by scuba divers. Red (*Gelidiopsis* sp.) and green (*Cladophora catenata*) macroalgae were plucked off the hard substrate. Species identification was mainly based on a previous survey [16].

### Sample processing and isotopic measurements

Soft tissues were extracted from sea anemones and corals and muscle tissues were extracted from the crab’s chelae and the sea snail’s foot. All the tissue and POM samples were dried at 60°C for a minimum of 24 h and ground into powder for further analyses. A portion of the powders was subject to acid treatment (1 N HCl) to remove any carbonate minerals in the tissues or POM. The acid treatment did not yield appreciable changes in the δ^15^N composition (0.1–0.2‰; N = 5), therefore, the effect of acid treatment on the isotopic composition was considered negligible. To obtain sufficient sample amounts for isotopic analysis, several individuals of zooplankton and epibenthic crustaceans, or muscle tissue from more than five individuals of sea snail *A*. *misera* at the same sites were pooled. However, the bottom POM from the NW transect was not sufficient for reliable nitrogen isotope determination and, therefore, is not reported.

Carbon and nitrogen isotope ratios were determined using an elemental analyzer (Thermo Flash EA) connected with a Thermo Finnigan MAT253 isotope ratio mass spectrometer at Institute of Oceanography, National Taiwan University. The analyzed results were reported in δ notation:

$$\delta X=\left(\frac{R_{sample}}{R_{standard}}-1\right)\times 1000\;(‰)$$ (Eq 1)

where X is ^13^C or ^15^N, R is ^13^C/^12^C or ^15^N/^14^N, and the standards for δ^13^C and δ^15^N are Vienna Pee Dee Belemnite and atmospheric N_2_ gas, respectively. The U.S. Geological Survey standard #40 (USGS40, L-glutamic acid with certified δ^13^C and δ^15^N values of −26.2 and −4.5‰, respectively), was used as the reference standard. The analytical error (1σ) derived from multiple analyses of the reference standards for both δ^13^C and δ^15^N is better than 0.2‰.

To precisely quantify the trophic position of the crab *X*. *testudinatus*, compound-specific nitrogen isotope analysis of amino acids was conducted for two individuals from the same site (SW0) with completely distinct δ^13^C (−13.9‰ versus −18.1‰) and δ^15^N values (+1.1‰ versus +9.0‰) to accurately estimate its trophic level (TL). The amino acid nitrogen isotope analysis was carried out at the Department of Biogeochemistry of the Japan Agency for Marine-Earth Science and Technology. The preparation of crab tissues for the amino acid isotope analysis mainly followed the procedures described by Chikaraishi et al. [22]. In brief, crab tissues were first hydrolyzed with 12 N HCl at 110°C for 24 h, followed by N-pivaloyl-isopropyl derivatization as suggested by Metges et al. [23]. The nitrogen isotope ratio of individual amino acids was determined by gas chromatography in line with a combustion oven and IRMS, and reported as the δ notation described previously. The analytical error (1σ) for the amino acid standards was better than 0.5‰.

### Modeling of isotopic mixing

An isotopic mixing model based on the Bayesian framework was used to quantify the contributions of potential energy sources to vent consumers by applying the open source Stable Isotope Analysis in R (SIAR) package [18]. The SIAR model incorporates the isotopic variabilities in food source and consumer, trophic discrimination factors (TDFs), and elemental concentrations of sources. This model implements the Markov Chain Monte Carlo method to produce simulations of plausible values of the proportional contributions that are consistent with the observed data. A total of 500,000 iterations were run to produce a simulation of the probability distributions for the relative source contributions of individual food sources. Due to the large intra-species isotopic variation (e.g., in crabs), the estimation of dietary compositions was performed for each individual rather than the population. The TDFs of both carbon and nitrogen isotopes (△δ^13^C and △δ^15^N), which exhibit great variability among taxonomic classes and feeding guilds [24,25], play a critical role in the Bayesian mixing model [26]; thus, we applied guild-dependent feeding TDFs for the isotopic mixing modeling herein. Accordingly, the applied TDFs of δ^13^C and δ^15^N were −0.41 ± 1.14‰ and +2.52 ± 2.50‰ for herbivores, and +0.91 ± 1.04‰ and +3.23 ± 0.41‰ for carnivores, respectively [24]. Decisions about food source selection were also influential in the modeling results and a fundamental knowledge of the system is a prerequisite for implementing the mixing model and interpreting the output data [27]. Therefore, potential diet items for each consumer species selected for the mixing model computation were referred to the feeding habit revealed in our plot of δ^13^C and δ^15^N values and described in the literature, e.g., for *E*. *contractus* [28] and *X*. *testudinatus* [16,19]. For primary consumers inhabiting pelagic and epibenthic zones, the energy sources were mainly contributed by POM. Therefore, we calculated the proportional contributions of peripheral seawater POM and vent POM.

### Estimation of the trophic level of *X*. *testudinatus*

While *X*. *testudinatus* was generally considered to occupy the top trophic rank in this ecosystem [19], two individuals with distinct, extreme bulk isotopic compositions were analyzed for the nitrogen isotope compositions of amino acids. The amino acid method has been used to estimate the trophic level of consumers without prior determination of the δ^15^N values of the primary producers [22,29]. By comparing the δ^15^N values of amino acids which exhibit a high degree of trophic enrichment (e.g., glutamic acid; aka trophic amino acids) with those showing little change in the δ^15^N values with trophic transfer (e.g., phenylalanine; aka source amino acids), the trophic level of one organism can be estimated based on the following equation [29]:

$${\mathrm{TL}}_{x/y}=\left(\delta^{15}N_{x}-\delta^{15}N_{y}-\beta_{x/y}\right)/\left(\Delta_{x}-\Delta_{y}\right)+1$$ (Eq 2)

where *x* and *y* represent trophic and source amino acids, respectively, β_*x/y*_ stands for the inherent isotopic difference between amino acids *x* and *y* in primary producers, and Δ_*x*_ and Δ_*y*_ indicate the ^15^N-enrichment factors for each trophic level for amino acids *x* and *y*, respectively. Among various amino acids, glutamic acid and phenylalanine have been widely accepted as the most useful pair to derive the precise estimation of trophic levels [29,30].

## Results

### Species distribution

During our visual survey through scuba diving, the vent crab *X*. *testudinatus* constituted the majority of the faunal abundance, followed by the sea anemones *Anthopleura* sp. and sea snails *A*. *misera*. More than 150 and 130 individuals of *X*. *testudinatus* and *Anthopleura* sp. were collected, respectively. Both species were distributed along the survey transect from the vent center to peripheral sites (100 m from the vent). The abundance of vent crabs decreased beyond 50 m from the vent, whereas sea anemones became more dominant at the periphery. Coral colonies (*Tubastraea* sp.) were mainly distributed within 50 to 100 m from the vent center, except for one colony observed at a site 10 m from the vent center in the NW transect. The sea snails were generally present with patches of green macroalgae (*C*. *catenata*) with *A*. *misera* being more abundant than *E*. *contractus*. The coverage of green and red macroalgae (*Gelidiopsis* sp.) was generally observed beyond 30 m from the vent center.

### Bulk carbon and nitrogen isotope compositions

**Fig 2:**
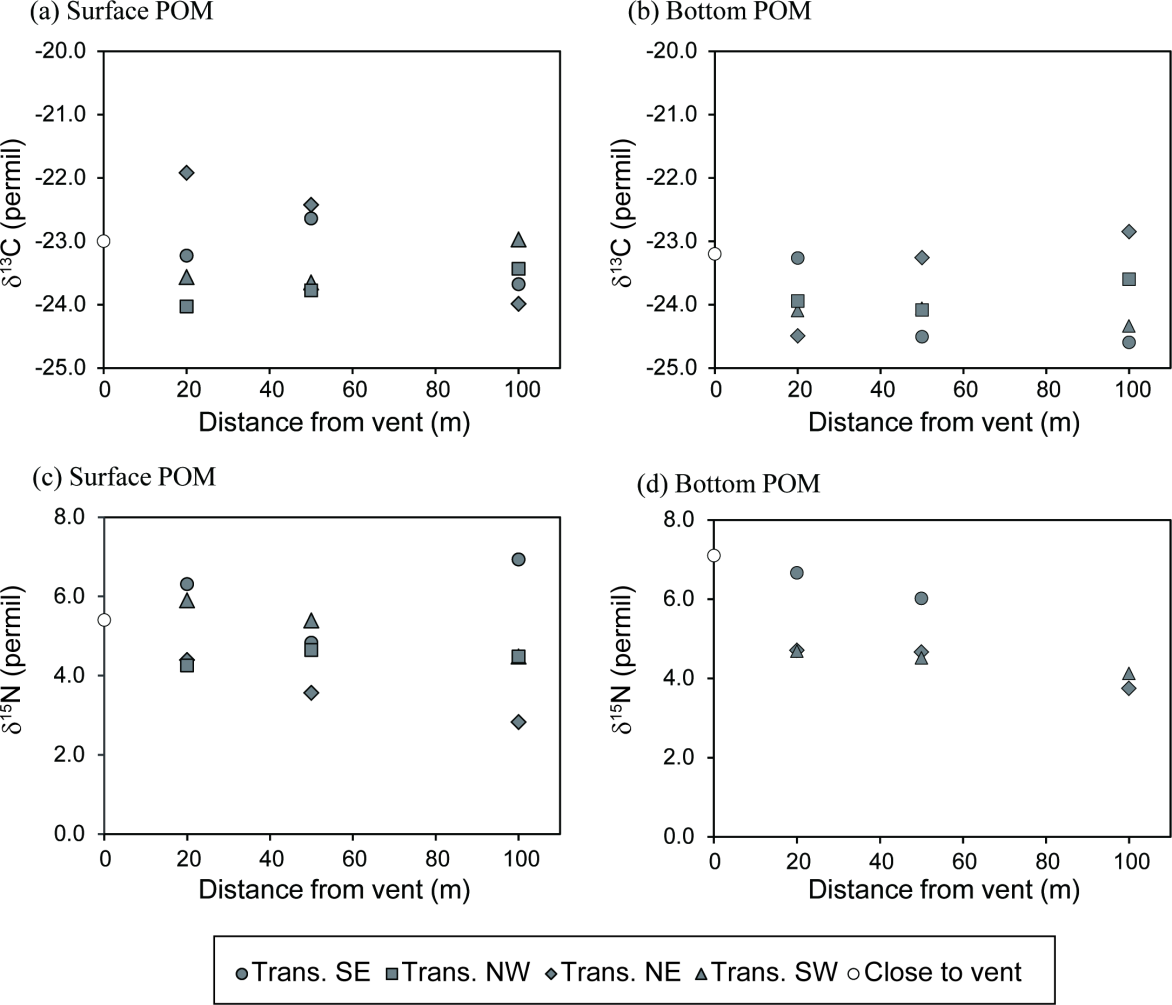
Spatial variations in δ^13^C and δ^15^N values for POM from each transect. (a, c) Isotopic compositions for surface water, (b, d) isotopic compositions for bottom water.

The δ^13^C and δ^15^N values of POM associated with the vent fluid were −18.2 ± 1.1‰ and −1.7 ± 0.4‰, respectively, and appear to be the most ^15^N-depleted end-member having a heavier carbon isotope composition among all potential food sources (Table 1). The δ^13^C and δ^15^N values of POM filtered from peripheral seawater ranged from −24.6 to −21.9‰ and from +2.8 to +7.1‰, respectively (Table 1). The variations in the δ^13^C value for surface seawater POM decreased with distance from the vent center (Fig 2A). No obvious trend in the δ^13^C variation was observed for the bottom POM samples (Fig 2B). The δ^15^N values of both surface and bottom POM showed large variations (2 to 4.3‰) among sampling sites (Table 1; Fig 2C and 2D). While the δ^15^N variations for bottom POM decreased with the distance from the vent center, the δ^15^N variations for surface POM exhibited no systematic trend. The δ^13^C values of red macroalgae (*Gelidiopsis* sp.) ranged between −32.9 and −22.2‰ (the most ^13^C-depleted carbon source), whereas the δ^15^N values ranged between +1.7 and +4.0‰. The δ^13^C and δ^15^N values of green macroalgae (*C*. *catenata*) ranged from −22.8‰ to −17.3‰ and from +3.7‰ to +5.7‰, respectively. Both red and green macroalgae exhibited extremely large δ^13^C variations across sampling sites (Table 2), yet no obvious trend was observed among the transects.

**Table 1:** Ranges (minimum and maximum) and mean values (± 1σ) of carbon and nitrogen isotope ratios for vent, surface, and bottom POM samples from all sampling transects.

Sampling site	δ^13^C (‰)				δ^15^N (‰)			
	N^1^	Min.	Max.	Mean ± std	N	Min.	Max.	Mean ± std
Vent fluid_2015	1	−17.4	−17.4	−17.4	1	−1.4	−1.4	−1.4
Vent fluid_2016	1	−19.0	−19.0	−19.0	1	−2.0	−2.0	−2.0
Mean ± std				−18.2 ± 1.1				−1.7 ± 0.4
Surface								
0 m	1	−23.0	−23.0	−23.0	1	+5.4	+5.4	+5.4
20 m	4	−24.0	−21.9	−23.2 ± 0.9	3	+4.3	+6.3	+5.0 ± 1.2
50 m	4	−23.8	−22.4	−23.1 ± 0.7	4	+3.6	+5.4	+4.6 ± 0.8
100 m	3	−24.0	−22.9	−23.5 ± 0.5	4	+2.8	+6.9	+4.7 ± 1.7
Bottom								
0 m^2^	1	−23.2	−23.2	−23.2	1	+7.1	+7.1	+7.1
20 m	4	−24.5	−23.3	−24.0 ± 0.5	3	+4.7	+6.7	+5.4 ± 1.1
50 m	4	−24.5	−23.3	−24.0 ± 0.5	3	+4.5	+6.0	+5.1 ± 0.8
100 m	4	−24.6	−22.9	−23.9 ± 0.8	1	+3.8	+3.8	+3.8
Mean ± std				−23.4 ± 0.7				+5.0 ± 1.1

^1^N is the sample number of the isotopic measurement.

^2^The “0m” stands for the closest location beside the vent center without visible influence of vent fluids.

**Table 2:** Taxonomic groups, trophic guilds, and mean (± 1σ) values of carbon and nitrogen isotope ratios for benthic flora and macrofauna.

Member	Taxon	N^1^	Trophic guild^2^	δ^13^C (‰)	δ^15^N (‰)
				Mean ± Std	Mean ± Std
Red macroalgae	*Gelidiopsis* sp.	15	PP	−25.3 ± 2.5	+2.9 ± 0.8
Green macroalgae	*Cladophora catenata*	9	PP	−20.1 ± 2.2	+4.6 ± 0.6
Epibenthic crustacean^3^	Amphipoda, Mysida, Euphausiacea	2	SF, G	−19.9 ± 0.1	+6.0 ± 0.6
Zooplankton	Copepoda	20	G	−21.0 ± 0.2	+6.1 ± 1.0
Coral	*Tubastraea* sp.	7	SF, PF	−20.3 ± 0.4	+8.8 ± 0.2
Sea anemones	*Anthopleura* sp.	51	SF, PF	−19.9 ± 0.4	+9.2 ± 0.3
Crab	*Xenograpsus testudinatus*	46	S/D	−17.2 ± 1.1	+8.2 ± 1.8
Sea snail	*Anachis misera*	13	S/D	−18.0 ± 0.7	+8.8 ± 0.3
	*Ergalatax contractus*	12	S/D, P	−17.1 ± 0.4	+8.7 ± 0.7

^1^N is the sample number of the isotopic measurement.

^2^Trophic guilds (PP: primary producer, SF: suspension feeder, G: grazer, PF: plankton feeder, S/D: scavenger/detritivores, P: predator).

^3^The isotope analysis of epibenthic crustaceans was performed for a pooled sample of various species.

The δ^13^C and δ^15^N values of zooplankton varied from −21.4 to −20.5‰ and from +4.2 to +7.6‰, respectively. Both the δ^13^C and δ^15^N values of zooplankton increased with size (Pearson’s r = 0.82 and 0.89, respectively, Table 3). The δ^15^N variations for small sized zooplankton (200–363 μm) were higher than those for large sized zooplankton from the same area. Zooplankton collected from venting areas possessed slightly but significantly higher mean δ^13^C values than those from non-venting areas (*p* < 0.01; Student’s *t*-test).

**Table 3:** Stable isotopic and dietary compositions of small epibenthic crustaceans and zooplankton from venting and non-venting areas.

	N^1^	δ^13^C (‰)	δ^15^N (‰)	POM^2^ (%)	vent POM^2^ (%)
Epibenthic crustaceans					
venting area	1	−19.8	+6.5	48	52
non-venting area	1	−20.0	+5.6	47	53
Zooplankton					
venting area					
> 2000 μm	2	−20.7 ± 0.3	+6.9 ± 0.5	57	43
1000–2000 μm	2	−20.7 ± 0.1	+6.7 ± 0.3	56	44
500–1000 μm	2	−20.9 ± 0.1	+6.0 ± 0.7	55	45
363–500 μm	2	−21.0 ± 0.1	+5.5 ± 0.6	54	46
200–363 μm	2	−21.2 ± 0.3	+5.3 ± 1.0	54	46
non-venting area					
> 2000 μm	2	−21.0 ± 0.1	+7.5 ± 0.1	62	38
1000–2000 μm	2	−21.2 ± 0.1	+6.5 ± 0.2	59	41
500–1000 μm	2	−21.1 ± 0.1	+5.9 ± 0.7	55	45
363–500 μm	2	−21.2 ± 0.2	+5.1 ± 1.4	54	46
200–363 μm	2	−21.3 ± 0.0	+5.3 ± 1.4	54	46

^1^N is the sample number of the isotopic measurement.

^2^POM: particulate organic matter in seawater; vent POM: vent-associated particulate organic matter. Their isotopic compositions are listed in Table 1.

**Fig 3:**
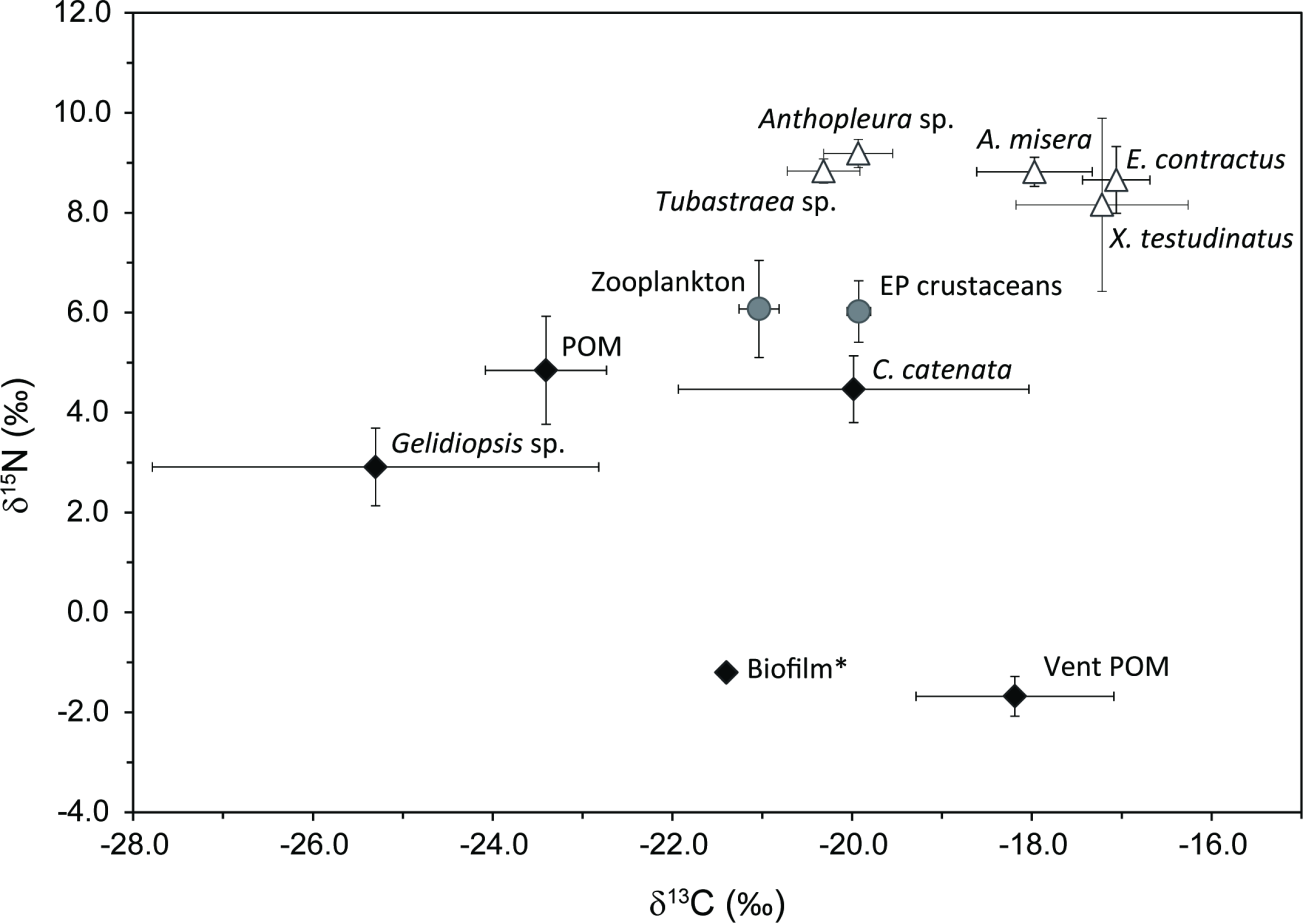
Plot of mean (± 1σ) δ^13^C versus δ^15^N values for major species. Black diamond, grey circles, and white triangles represent primary producers, primary consumers, and higher order consumers, respectively. The * symbol indicates isotopic compositions of biofilm on *X*. *testudinatus* adopted from Wang et al. [19].

The epibenthic crustaceans, which were mainly composed of amphipod, mysida, Euphausiacea, and unidentified species, had δ^13^C and δ^15^N values ranging from −20.0 to −19.8‰ and +5.6 to +6.5‰, respectively. Their δ^13^C values were slightly higher than those for zooplankton (Table 3). The mean δ^13^C values of zooplankton and epibenthic crustaceans were higher than those for averaged seawater POM by 2.4 and 3.5‰, respectively. However, the difference in the mean δ^15^N value between these two primary consumers and seawater POM was only *ca*. 1‰ (Fig 3).

**Fig 4:**
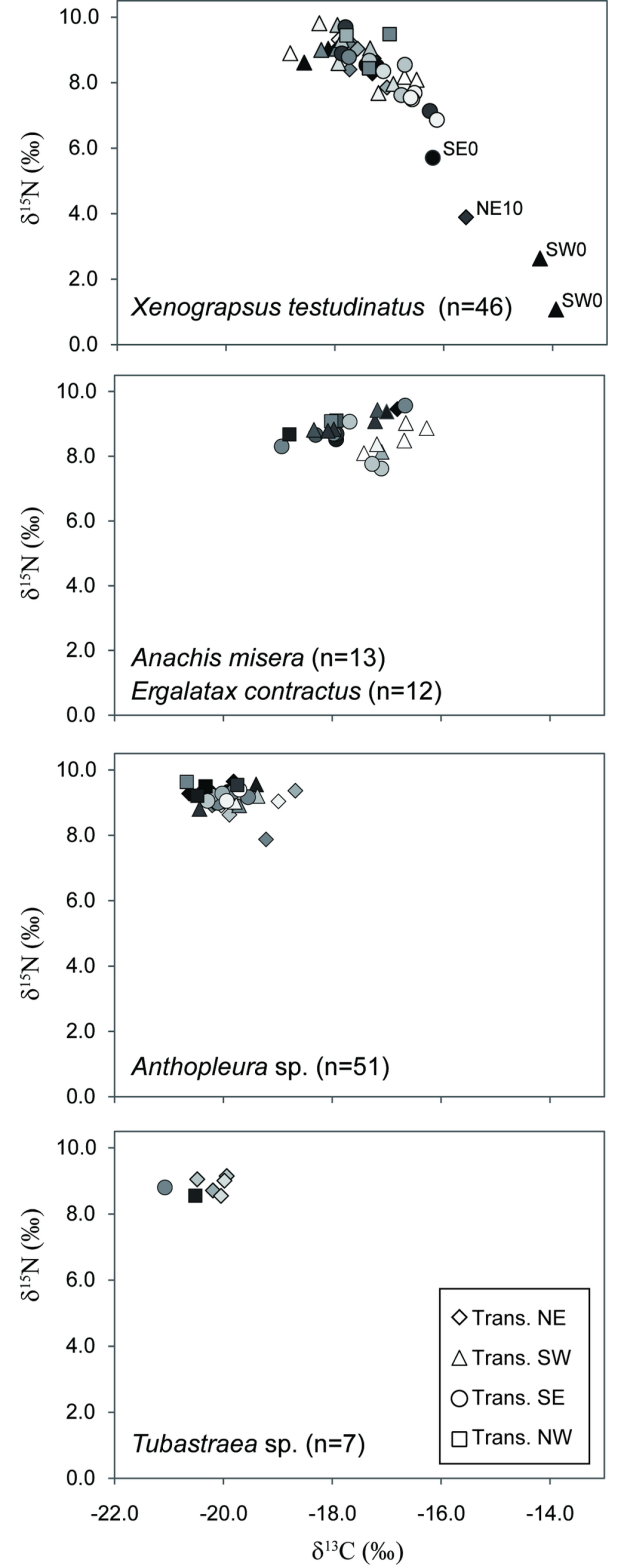
Plots of δ^13^C versus δ^15^N values for crab (*X*. *testudinatus*), sea snails (*A*. *misera* and *E*. *contractus*), sea anemones (*Anthopleura* sp.), and coral (*Tubastraea* sp.) individuals. Different grey levels indicate different distances from the vent center with black and white symbols representing samples from the vent center and 100 m, respectively.

The tissue δ^13^C and δ^15^N values of benthic macroinvertebrates ranged from −21.4 to −13.9‰ and from +1.1 to +9.8‰, respectively (Table 2). The crab *X*. *testudinatus*, and sea snails *A*. *misera* and *E*. *contractus* identified as scavenger/detritivores shared similar mean δ^13^C and δ^15^N values of *ca*. −17 and +8‰, respectively. Detritivores revealed obviously large isotopic variations in terms of spatial scale. In particular, the variations in the δ^13^C and δ^15^N values of vent crabs even reached +4.9 and +8.7‰, respectively (S1 Table). Such a large variation arises from the fact that a small number of the vent crabs collected from the 0 to 10 m-sites were characterized by extremely high δ^13^C and low δ^15^N values that were distinct from the majority of the data (Fig 4). The two species of sea snail were similar in their isotopic characteristics and revealed relatively smaller conspecific isotopic variations than the vent crabs. Suspension feeders showed small isotopic variations across sampling sites. The δ^13^C and δ^15^N values of corals, *Tubastraea* sp., ranged from −21.1 to −19.9‰ and from +8.6 to +9.2‰, respectively. Sea anemones (*Anthopleura* sp.) exhibited slightly larger isotopic variations than corals with the δ^13^C and δ^15^N values varying from −20.7 to −18.7‰ and from +7.9 to +9.7‰, respectively. Corals and sea anemones, which are classified as carnivorous suspension feeders, exhibited comparable δ^15^N values yet obviously more depleted δ^13^C values (−19.9 to −20.3‰) than scavenger/detritivores. No clear spatial trend in the isotopic compositions was observed for most of the macroinvertebrate species, except for the vent crabs, whose isotopic compositions show a clear negative correlation (Fig 4).

### Amino acid nitrogen isotopes and the trophic position of vent crabs

The averaged δ^15^N values of triplicate measurements of glutamic acid for two individuals were +8.9 ± 0.3‰ and +14.2 ± 0.3‰ (Table 4). The corresponding δ^15^N values of phenylalanine were −1.0 ± 0.4‰ and −5.9 ± 0.4‰. The calculated trophic positions were 2.5 for both of the analyzed individuals.

**Table 4:** Nitrogen isotope compositions of glutamic acid (Glu) and phenylalanine (Phe) based on triplicate analyses and the calculated trophic position (TP) for two vent crabs.

	Amino Acid	δ^15^N (‰)					Average TP
		#1	#2	#3	Mean	SD	
Crab #1	Glu	+14.5	+14.0	+14.0	+14.2	0.3	2.54
	Phe	−1.3	−0.5	−1.0	−1.0	0.4	
Crab #2	Glu	+8.6	+8.7	+9.2	+8.9	0.3	2.50
	Phe	−6.1	−5.4	−6.2	−5.9	0.4	

SD: standard deviation.

### Dietary compositions of vent invertebrates from the mixing model

**Fig 5:**
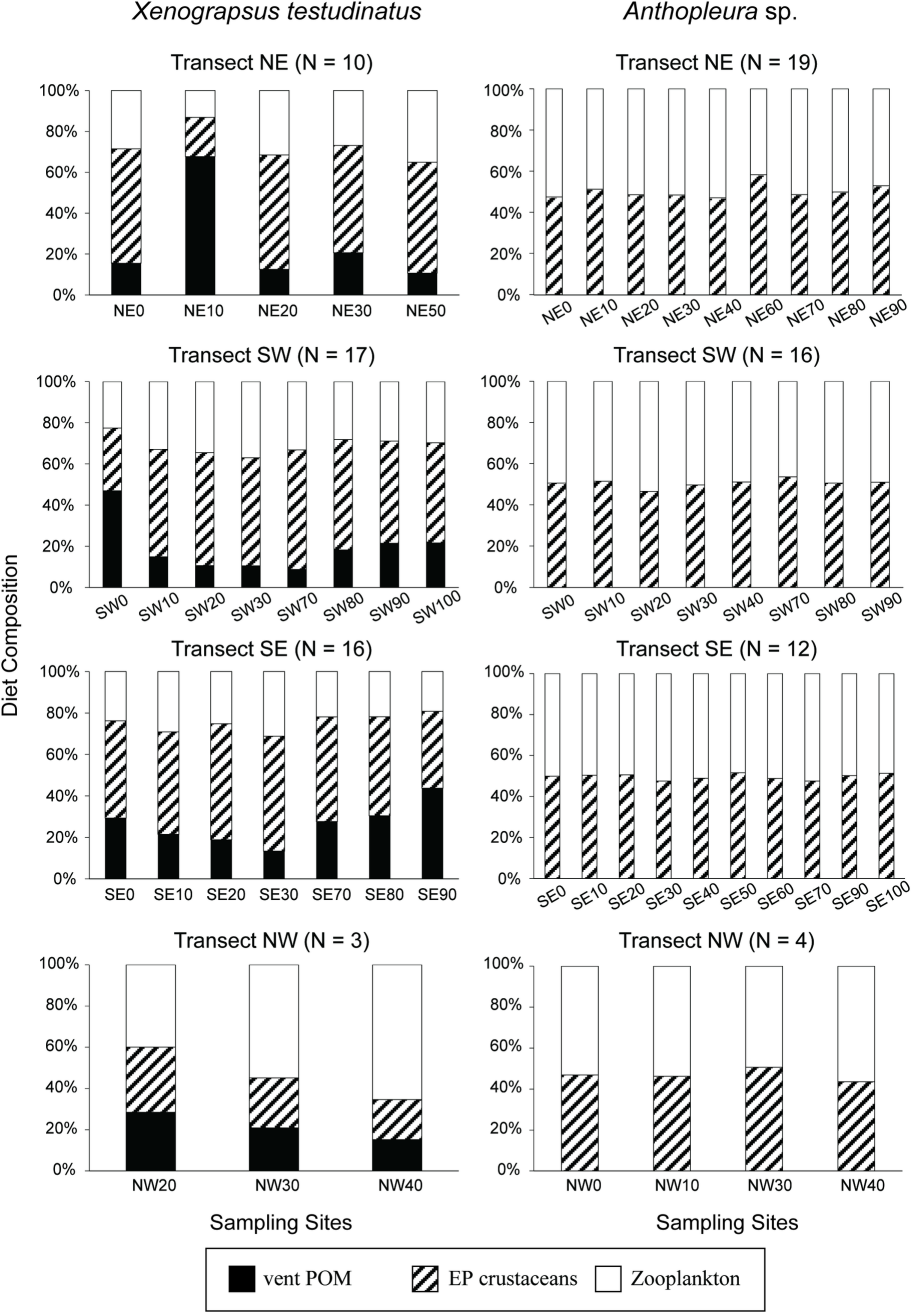
Mean diet compositions of vent crab (*X*. *testudinatus*) and sea anemones (*Anthopleura* sp.) from each sampling site (only one individual in each site of the NW transect). EP crustaceans: epibenthic crustaceans.

About half of the diet of epibenthic crustaceans was contributed by vent POM (52–53%) and seawater POM (47–48%) (Table 3). A total of 38–46% of the zooplankton’s diet was from vent POM. Different size zooplankton were not obviously different in their dietary composition (1σ = 0.02). Both epibenthic crustaceans and zooplankton showed little difference (less than 5%) in their dietary compositions between venting and non-venting areas. While zooplankton showed δ^13^C values distinct from those of crab (mean Δδ^13^C = 3.8‰, Fig 3), it contributed a proportion of 26 ± 9% to the crab’s diet on average (Fig 5). The epibenthic crustaceans, which had δ^13^C values more closely resembling those of the crabs than the zooplankton (Fig 3), constituted 50 ± 13% of the crab’s diet. Additionally, the extremely ^13^C-enriched and ^15^N-depleted values for some crabs (Fig 4) suggest the involvement of an additional pool with a distinct carbon and nitrogen isotopic composition. Of all the candidates, the vent POM characterized by the highest δ^13^C and lowest δ^15^N values appears to be the most plausible source. Overall, the computation yielded a contribution from vent POM to the crab’s total diet 6% to 87% (mean ± 1σ = 24 ± 20%) (S1 Table), with the largest dependence on vent POM for crabs collected from the 0–10 m sites (e.g., NE10 and SW0).

**Fig 6:**
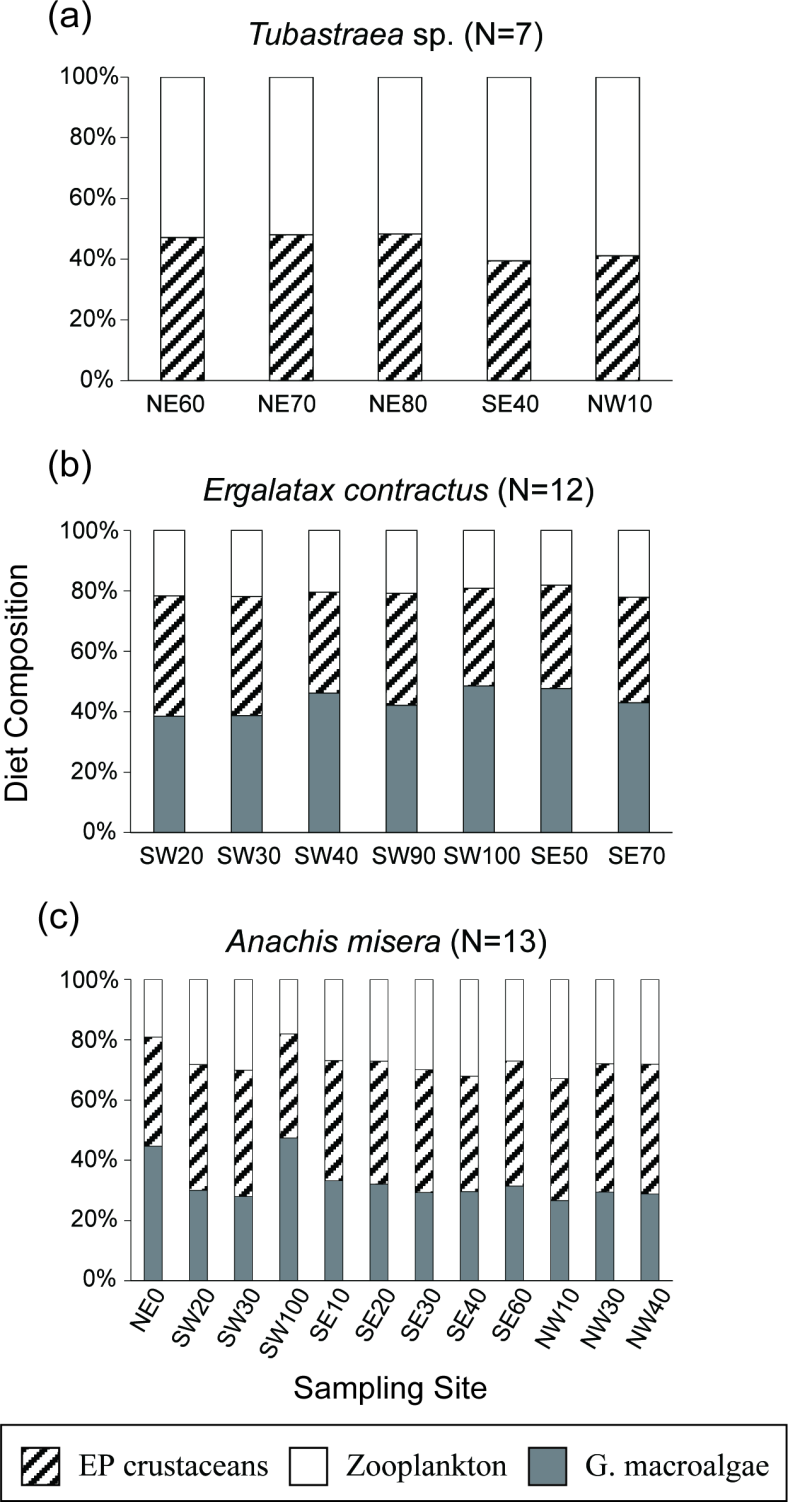
Mean diet compositions of coral (*Tubastraea* sp.) and sea snails (*A*. *misera* and *E*. *contractus*) from each sampling site. EP crustaceans: epibenthic crustaceans; G. macroalgae: green macroalgae.

Sea anemones exhibited δ^13^C values similar to epibenthic crustaceans (mean Δδ^13^C < 0.1‰) and zooplankton (mean Δδ^13^C = 1.1‰). Their computed dietary composition was highly consistent across all transects, with equal ingestion of zooplankton and epibenthic crustaceans (ca. 50 ± 3%; Fig 5). The coral *Tubastraea* sp. showed an isotopic composition similar to sea anemones (Δδ^13^C = 0.4%; Δδ^15^N = 0.4%), and their diet compositions were not significantly different among coral colonies (54 ± 4% for zooplankton and 46 ± 4% for epibenthic crustaceans; Fig 6A). Both the sea snails *A*. *misera* and *E*. *contractus*, showed overlapping isotopic values with crabs. Their major food items were composed of green macroalgae (44 ± 6% for *E*. *contractus* and 32 ± 6% for *A*. *misera*), corpses of epibenthic crustaeans (35 ± 4% for *E*. *contractus* and 40 ± 3% for *A*. *misera*), and zooplankton (21 ± 2% for *E*. *contractus* and 27 ± 4% for *A*. *misera*) (Fig 6B and 6C). Both *E*. *contractus* and *A*. *misera* exhibited negligible spatial variation in dietary compositions.

## Discussion

### Stable isotope characteristics of potential food sources

Our data revealed three major groups of isotopically distinct food sources in the KST vent field: vent POM, seawater POM, and green benthic macroalgae (Fig 3). The disparity in the isotopic compositions of these organic pools strongly reflected different metabolic pathways of energy production [31] and allowed us to evaluate their respective contributions to the food web. Although biofilms on the seafloor or biotic surfaces have been recognized as a common feature of hydrothermal vent ecosystems, they are usually composed of a range of constituents, such as diatoms, algae–bacteria, and bacteria, with various isotopic compositions [10]. The only isotopic data for biofilms in the KST vent field showed an extremely low δ^15^N value (−1.2‰) but a mid-ranged δ^13^C value (−21.4‰) [19], which suggested it had little contribution to benthic consumers (with high δ^13^C and δ^15^N values).

Among these food sources, the vent POM, which is primarily composed of chemoautotrophic microbial biomass [32,33], was particularly enriched in ^13^C and depleted in ^15^N (δ^13^C = −18.2 ± 1.1‰, δ^15^N = −1.7 ± 0.4‰). This distinct isotopic composition is rarely reported and is comparable with those speculated for free-living chemosynthetic bacteria in deep-sea hydrothermal vents [2,11,34,35]. The δ^13^C values of autotrophic organisms are strongly dependent on (1) isotopic fractionation during biosynthetic processes, and (2) the isotopic composition of inorganic carbon sources [31,36]. In the hydrothermal vent fields, the reductive tricarboxylic acid cycle (rTCA) used by chemoautotrophic bacteria, such as Epsilon-Proteobacteria, the thermophilic Aquificales order, and the archaeal Thermoproteaceae family, has been reported to be the predominant carbon fixation pathway [37,38]. The rTCA synthesizes acetyl-CoA from CO_2_ and tends to yield significantly less carbon isotopic fractionation (Δδ^13^C_cell_ ranged from −3.3 to −13.3‰) than the Calvin cycle (light-independent reaction of photosynthesis) and the reductive acetyl-CoA pathway [31,39], thus producing ^13^C-enriched organic carbon. In the bottom sediments of the KST vent area and crab tissues, 16S ribosomal RNA gene amplicon pyrosequencing demonstrated the predominance of Epsilon-Proteobacteria (*Sulfurovum and Sulfurimonas)* [40–42], most of which have been proposed to use the rTCA cycle as the major carbon fixation pathway [38]. Apparently, the enrichment of ^13^C in vent POM could also be derived from dissolved inorganic carbon (DIC) with a higher δ^13^C value. However, the carbon isotopic compositions of CO_2_ gas released from venting fluids in the KST vent field, ranging from −8.2 to −5.5‰ [43], seems to preclude a source of ^13^C-enriched DIC for chemoautotrophs in the KST venting fluids. The distinct high δ^13^C value of vent POM obtained in this study has likely originated from the chemoautotrophic microbial populations using the rTCA cycle as the major biosynthetic pathway and exploiting vent DIC as the primary carbon source.

The vent POM is also extremely ^15^N-depleted. Bacterial mats in hydrothermal vent fields (sulfide oxidizing bacteria and other unidentified forms) have been widely and consistently characterized by low δ^15^N values (*ca*. −9.6 to +1.6‰) [2,19,44,45] compared to photosynthesis-based food sources (δ^15^N ≈ +4 to +8‰ [45,46]). Such a ^15^N-depleted isotope composition can likely be attributed to the pronounced bacterial NH_4_^+^ assimilation by autotrophic microorganisms [44], which can generate a nitrogen isotopic fractionation an order of magnitude larger than N_2_ fixation or NO_3_^-^ assimilation (*ca*. 20‰ versus 0 to 5‰ [47,48]). In this way, the biomass formed through ammonium assimilation would be depleted in ^15^N. Overall, the interpretation of the origin of vent POM enriched in ^13^C and depleted in ^15^N is mutually coherent. We also note that only a few measurements might not adequately address the dynamic nature of hydrothermal environments. Thus, increasing efforts to repeatedly sample and analyze vent POM would facilitate the validation of temporal variations of chemosynthetic sources.

The isotopic compositions of seawater POM obtained in this study resembled those for marine planktonic phototrophs (δ^13^C values between −24.0 and −19.0‰ and δ^15^N values between +4.0 and +8.0‰ [46]). These isotopic values were also comparable with those for POM collected from adjacent areas (e.g., δ^13^C values between −23.0 and −19.9‰ and δ^15^N values between +3.7 and +4.3‰ for POM from the outer shelf of the East China Sea [49] and δ^13^C values between −24.8 and −21.1‰ and δ^15^N values between +3.0 and +4.5‰ for POM in sediment trap and sediments from the Okinawa Trough [50, 51]). The chemosynthetically-derived production characterized by the ^13^C-enriched and ^15^N-depleted isotopic signature was barely observed in the seawater POM, suggesting restricted influence of the vent fluid on the surrounding water mass. However, the high spatial variations in the δ^13^C value of POM at four 20-m sites (Fig 2A) suggest various degrees of mixing between seawater and buoyant vent fluid along different transects.

Both green and red macroalgae exhibited large variations in δ^13^C value (> 5.0‰). Their δ^13^C values still fell in an extremely large range reported for worldwide marine macroalgae (−3.0 to −35‰) [52,53]. Such a considerable isotopic variation was inferred to be attributed to the variable concentrations and forms of DIC used, *in situ* temperature, and taxonomy [53]. However, it is still difficult to thoroughly explain this variation [54].

### Isotope characteristics and dietary compositions of vent consumers

Our isotopic evidence combined with the modeling results demonstrated a widespread contribution of vent POM to the food web. In particular, a large disparity of δ^13^C values between most benthic invertebrates and seawater POM was observed (Fig 3). For instance, the mean δ^13^C values of the sea anemones *Anthopleura* sp. and crab *X*. *testudinatus* were greater than seawater POM by 3.5 and 6.1‰, respectively. Even zooplankton, which is well acknowledged as the primary consumer, showed distinct δ^13^C values yet similar δ^15^N values compared to seawater POM. Such decoupled isotopic discrimination was unlikely to be solely attributed to the trophic enrichment. Instead, it strongly indicated a mixed diet of seawater POM and an end-member enriched in ^13^C and depleted in ^15^N, i.e., the vent POM for consumers in the ecosystem. We discuss the dietary compositions for the major macrofauna below.

#### Zooplankton and epibenthic crustaceans

Our data indicated that zooplankton and epibenthic crustaceans acted as fundamental consumers mediating the transfer of both chemosynthetic and photosynthetic energy to the higher trophic levels. Epibenthic crustaceans, including mysids, amphipods, and krill, which have never been reported in other shallow-water vent ecosystems, had δ^13^C values closer to the vent POM than zooplankton had. This isotopic data combined with the mixing modeling indicated that vent POM constituted about half of the diet for epibenthic crustaceans, a magnitude greater than that for zooplankton (Table 3). The higher dietary consumption of vent POM for epibenthic crustaceans likely resulted from their greater access to the bacterial biomass associated with vent fluids.

In shallow-water vents, chemosynthesis takes place not only in the bottom water layer but also near the sea surface, and even spreads over the surface layer due to the warmth and buoyancy of hydrothermal fluids [6,10]. Although the extent of chemosynthesis in surface water is rarely quantified, our results indicating substantial contribution of vent POM to the zooplankton diet (38 to 46%) strongly suggests that even the pelagic consumers can feed on chemosynthetic production discharged to surface water. As an important component of shallow-water vents, zooplankton may accelerate the energy and material exchange between the pelagic and benthic subsystems *via* diel vertical migration (active transport) and passive sinking of detrital materials [10,55], thus enabling strong benthic−pelagic coupling in coastal shallow-water hydrothermal vents. Moreover, since zooplankton can be passively transported by currents and tides [56], lateral export of chemosynthetic energy from the venting area to the adjacent open ocean would be highly possible.

#### Benthic macrofauna

Our study revealed distinct carbon isotope compositions among consumers affiliated with different feeding guilds. The disparity in δ^13^C values between suspension feeders and scavenger/detritivores reached *ca*. 3‰, yet the differences in the δ^15^N value were less than 1‰. Such discordance between different isotopes has also been observed for deep sea counterparts [2,19,57], reflecting the existence of multiple food sources with distinct isotopic characteristics and the diverse feeding selectivity of consumers in hydrothermal systems [2].

Carbon isotope compositions for carnivorous suspension feeders, *Tubastraea* sp. and *Anthopleura* sp., were close to those for zooplankton and epibenthic crustaceans, whereas the difference in their nitrogen isotopic values approached 3‰. The isotopic pattern suggests a close prey–predator relationship between suspension feeders and these fundamental species. The mixing model further demonstrated almost equal dietary proportions from zooplankton and epibenthic crustaceans across all the sampling sites for both corals and sea anemones (Figs 5 and 6A).

The vent crab *X*. *testudinatus*, which is categorized as an omnivorous generalist [18,19,58], exhibited large individual variations in both δ^13^C and δ^15^N values, resembling the pattern of *Xenograpsus ngatama*, which inhabits the shallow hydrothermal vents off the South Tonga Arc [35]. Based on gut content analysis and field observations, a previous study concluded that the major dietary source for vent crabs is sunken zooplankton toxified by large amounts of hydrogen sulfide released during a period of strong venting [18]. However, our isotopic data revealed that > 80% of crab individuals exhibited higher δ^13^C values than those of zooplankton by more than 3‰, a magnitude exceeding the conventional isotopic fractionation (*ca*. 1‰ [59]) associated with feeding solely on zooplankton (Fig 3 and S1 Table). Similarly, the differences in the δ^15^N values between 65% of crabs and zooplankton were less than 3.4‰ (conventionally representative of one trophic level [60]) (Fig 3 and S1 Table). Both carbon and nitrogen isotope compositions preclude zooplankton as the main dietary source of crabs. The highly variable or extreme isotopic compositions of crabs suggest their preference for incorporating a diet source with isotopic compositions that are enriched in ^13^C and depleted in ^15^N. Among all the investigated organisms or entities, vent POM appears to be the most plausible candidate to explain the isotopic variations of crab tissues (Figs 3 and 4).

Using vent POM as an end member, our modeling computation indicated that the vent POM could contribute 9−67% of crab’s diet on average for different sites (Fig 5). In particular, some crab individuals inhabiting areas near the vent even showed overwhelming use (up to 87%; SI Table) of chemosynthetically-derived organic carbon, suggesting the high palatability and availability of vent POM for vent-obligate species. In addition, this phenomenon verified the observation of access to vent primary production by species colonizing under harsher conditions [61]. A previous study demonstrated the predominance of the fatty acid MUFA (n-7) series in the crab’s midgut [58]. In addition, bacterial lipids speculatively derived from autotrophic sulfur-oxidizing bacteria have been proposed to be incorporated or utilized by a bivalve *Solemya velum* symbiosis in sulfide-rich sediments [62]. Aside from the ingestion of vent POM, the mixing model also indicated that epibenthic crustaceans play a vital role in the crab’s diet, coinciding with the existence of highly active chitinolytic enzymes in the crab’s midgut glands [58]. Our estimate of the crab’s trophic position based on amino acid nitrogen isotope compositions revealed a consistent TL of 2.5 for the vent crabs regardless of the large variations in δ^15^N values. This result further supports the observation of the crab’s mixed diet of fundamental consumers (TL = 2) and chemosynthetic primary production (TL = 1). Although the green macroalgae sampled from some sites also exhibited high δ^13^C values, a previous study revealed common yet very limited use of the green algae Ulvophyceae by *X*. *testudinatus*, based on its 18S ribosomal DNA libraries of stomach and gut content [63]. The fact that δ^15^N values for a portion of the crab individuals were even lower than those of the macroalgae verified the limited contribution of macroalgae to the crab’s diet.

The sea snails *A*. *misera* and *E*. *contractus* are common species distributed along the shallow coastal areas of Taiwan. In comparison with the vent crabs, *A*. *misera* and *E*. *contractus* had similar isotopic compositions (Table 2 and Fig 3). Unlike the vent crabs, sea snail individuals with highly ^13^C-enriched and ^15^N-depleted signatures were absent from all sites, and the isotopic variations were much smaller. Accordingly, the relatively enriched ^13^C signals of sea snails could be mostly attributed to the ingestion of the green macroalgae *C*. *catenata*. Our mixing modeling results demonstrated that green macroalgae, which acts as a palatable food and shelter for gastropods in a wide variety of aquatic habitats [64], contributed 28% to more than half of the sea snails’ diet. If the photosynthetic contributions in other diets (zooplankton and epibenthic crustaceans) are also considered, sea snails acquire a total of 60–80% of their diet from photosynthetic production and less than 30% of their diet from chemosynthetic production (S1 Table).

Vent-associated species have been reported to possess distinctly lower δ^15^N values compared to non-vent species [1,2,4,34,65]. This δ^15^N signature could be indicative of a vent origin for dietary nitrogen and thus useful to identify vent-dependent fauna [1]. In comparison with other detritivorous decapods inhabiting the non-vent upper bathyal regions off KST (e.g., *Hymenopenaeus equalis* and *Heterocarpus sibogae*) [19], the δ^15^N values of the vent crabs *X*. *testudinatus* reached below *ca*. 2‰. Even the carnivorous sea anemones *Anthopleura* sp., which possessed the highest δ^15^N values among all species (mean δ^15^N = 9.2 ± 0.3‰), were characterized by δ^15^N values lower than its congeneric species, the algal symbiont-host *Anthopleura xanthogrammica* inhabiting Pacific intertidal areas [66]. The consistent depletion in ^15^N for consumers (< 10.0‰ for all individuals) in the KST vent system further supports the extensive consumption of chemosynthetic production as a complement to a photosynthesis-based diet in shallow-water hydrothermal vent ecosystems.

The stable isotope mixing model based on the Bayesian framework provided a quantitative insight into both chemosynthetic and photosynthetic contributions in a shallow-water hydrothermal food web. However, such a mixing model has been demonstrated to be sensitive to discrimination factors [26,27]. Variability in the discrimination factor may result in significantly different dietary proportions. In the present study, a set of TDFs that takes feeding guilds into account was introduced to the modeling. The approach reduces the possible bias in the estimate of diet composition. Besides, large differences between the isotope compositions of consumers and diets can further decrease the uncertainty introduced during the selection of specific TDFs [67]. With δ^13^C and δ^15^N values distinct from that of zooplankton, epibenthic crustaceans, and vent POM, the dietary compositions of the vent crabs should be reliable. Slightly greater uncertainty may arise in the diet determination for corals and sea anemones due to the relatively small difference in δ^13^C values between the consumers and food sources.

Since several vent crab individuals exhibited carbon isotope composition even heavier than that of the measured vent POM, a hypothetical vent POM with a δ^13^C value greater than that of the heaviest crab individual might be needed to account for the crab’s diet. With this potential diet source, the contribution of hypothetical vent POM to the diet of various consumers would decrease accordingly. To quantitatively constrain this source composition, we first consider a scenario where the trophic position of the crab with the heaviest carbon isotopic composition is 2.5 and the other food sources for this crab were a mixture of zooplankton and epibenthic crustaceans. The simple mass balance generates δ^13^C and δ^15^N values of −8.0‰ and −9.9. for the hypothetical vent POM, respectively. Using these compositions, the SIAR simulation yielded that 15–22% of the diet for zooplankton and small epibenthic crustaceans, and 48% of the diet for the heaviest crab are derived from the hypothetical vent POM. Alternatively, if the peculiar crab with the greatest δ^13^C value relies completely on the hypothetical vent POM (trophic position is 2), the δ^13^C and δ^15^N values for the hypothetical vent POM would be −13.5‰ and −1.4‰, respectively. Using these compositions, the SIAR simulation yielded that 28–40% of the diet for zooplankton and small epibenthic crustaceans and 79% of the diet for the heaviest crab are derived from the hypothetical vent POM. Regardless of which scenario, the vent POM still contributes significantly to the diet of a fraction of crab individuals and other primary consumers. Apparently, an accurate estimation of the vent POM contribution relies on a fully evaluation of the isotopic composition of vent POM.

### Trophic structure and energy flow

In deep-sea hydrothermal systems, the transfer of energy from plume-associated chemosynthetic production to higher trophic levels, particularly to the pelagic populations, was rarely observed due to the lack of sampling at a fine depth resolution [68] and biomarker evidence indicative of trophic or diet relationships [7]. With the isotopic approach described herein, this study clearly demonstrated various contributions of vent-derived chemosynthetic production to consumers inhabiting the KST hydrothermal field. In this system, species neither solely fed on photosynthetic production nor exclusively depended on chemosynthetic energy (except for a few crabs feeding on a high proportion of vent POM). All the sampled consumers exploited both chemosynthetic and photosynthetic carbon pools to varying degrees.

The prey–predator relationship among macrobenthic consumers was weak, as evidenced by the highly overlapping δ^15^N values (Table 2 and Fig 3). Such an isotopic pattern with a small range of δ^15^N values accompanied with a wide range of δ^13^C values for vent fauna indicates species partition of food resources between isotopically different carbon sources rather than prey–predator trophic relationships [61]. Previous studies widely recognized productivity as the most important factor affecting food web structure [69]. Particularly, food chain length (FCL) has been suggested to lengthen with increasing productivity [70,71]. Compared with the deep-sea hydrothermal vents, the shallow-water counterpart receives an extra input of photosynthetic production in addition to chemosynthetic production, thereby likely exhibiting a longer FCL. However, the KST hydrothermal ecosystem revealed an FCL (2–3 levels) shorter than or equal to those in deep-sea hydrothermal vents (e.g., 2–3 levels for the Mohns Ridge [44], 2.5–3.5 levels for the Marianas Trough and Hanging Gardens hydrothermal vents [34], and 4 levels for the Mid Atlantic Ridge [44]), suggesting that the productivity was likely not the determining factor. A previous study examined four vent assemblages from the Guaymas Basin and demonstrated that hydrothermal ecosystems subject to high fluid fluxes yielded strong environmental stress, facilitating the colonization of a few resistant taxa [72]. Under these circumstances, the dynamic and extreme environmental conditions offset and even overwhelm the beneficial effect of extra primary production to lengthen the FCL, instead leading to an overall reduction of food web complexity [44, 72]. Therefore, the weak prey–predator links observed for the shallow-water hydrothermal ecosystems could be attributed to the unique environmental context rather than productivity [61,73].

### Summary

This study provides the first quantitative evaluation of chemosynthetic and photosynthetic contributions to the shallow-water hydrothermal vent food web *via* a thorough isotopic survey of potential food sources and vent-related species with knowledge-based numerical modeling of mixed diets. Our results indicated that the chemosynthetic and photosynthetic productions contributed nearly equally to the food web. Both zooplankton and epibenthic crustaceans acted as an important trophic mediator transferring basic food sources to the upper trophic levels. While vent-obligate crabs are omnivorous, acquiring energy from all available food sources, few of them exhibited the potential to proliferate exclusively on chemoautotrophic energy. Given that hydrothermal vents are characterized by highly temporal variations in habitat conditions [61] and isotopic compositions of local autotrophs [2,4], we recommend a longer-term observation of the isotopic variation of end members and consumers to provide more comprehensive insight into this distinctive trophic regime and the energy exported to adjacent ecosystems.

## Supporting information

S1 Table Carbon and nitrogen isotopic compositions and computed dietary compositions of biological samples at each sampling site. (DOCX)
