# Supplementary material for: Trophic structure and energy flow in a shallow-water hydrothermal vent: Insights from a stable isotope approach
Source: PLoS One. 2018 Oct 17;13(10):e0204753. doi: 10.1371/journal.pone.0204753 (PMC6192584; doi:10.1371/journal.pone.0204753)
Supplement: S1 Table — (DOCX) [file pone.0204753.s001.docx]

**S1 Table. Carbon and nitrogen isotopic compositions and computed dietary compositions of biological samples at each sampling site. Standard deviations are given in parentheses**

| Taxa | N^1^ | Mean  δ^13^C  (‰) | Mean  δ^15^N  (‰) | sampling sites | δ^13^C  (‰) | δ^15^N  (‰) | Dietary items | | | |
| --- | --- | --- | --- | --- | --- | --- | --- | --- | --- | --- |
|  |  |  |  |  |  |  | Vent  POM (%) | Zooplankton (%) | Epibenthic crustaceans (%) | Green macroalgae (%) |
| *Gelidiopsis* sp. | 15 | －25.3 (2.5) | ＋2.9 (0.8) | NE30 | －27.8 | ＋3.4 | - | - | - | - |
|  |  |  |  | NE40 | －23.9 | ＋1.9 | - | - | - | - |
|  |  |  |  | NE50 | －26.2 | ＋3.3 | - | - | - | - |
|  |  |  |  | SW20 | －23.9 | ＋2.1 | - | - | - | - |
|  |  |  |  | SW40 | －24.3 | ＋2.3 | - | - | - | - |
|  |  |  |  | SE10 | －24.0 | ＋3.7 | - | - | - | - |
|  |  |  |  | SE30 | －25.5 | ＋3.7 | - | - | - | - |
|  |  |  |  | SE40 | －22.2 | ＋2.1 | - | - | - | - |
|  |  |  |  | SE50 | －24.9 | ＋3.3 | - | - | - | - |
|  |  |  |  | SE60 | －25.2 | ＋4.0 | - | - | - | - |
|  |  |  |  | SE70 | －25.3 | ＋1.7 | - | - | - | - |
|  |  |  |  | SE80 | －23.0 | ＋2.5 | - | - | - | - |
|  |  |  |  | SE100 | －25.7 | ＋4.0 | - | - | - | - |
|  |  |  |  | NW0 | －32.9 | ＋3.0 | - | - | - | - |
|  |  |  |  | NW10 | －24.8 | ＋2.9 | - | - | - | - |
| *Cladophora catenata* | 9 | －20.1 (2.2) | ＋4.6 (0.7) | NE30 | －22.8 | ＋4.2 | - | - | - | - |
|  |  |  |  | NE40 | －24.2 | ＋4.5 | - | - | - | - |
|  |  |  |  | NE60 | －18.5 | ＋4.0 | - | - | - | - |
|  |  |  |  | NE60 | －19.0 | ＋4.7 | - | - | - | - |
|  |  |  |  | SW80 | －17.3 | ＋3.7 | - | - | - | - |
|  |  |  |  | SE30 | －20.7 | ＋5.0 | - | - | - | - |
|  |  |  |  | SE80 | －19.8 | ＋5.4 | - | - | - | - |
|  |  |  |  | SE90 | －19.1 | ＋4.5 | - | - | - | - |
|  |  |  |  | SE100 | －19.8 | ＋5.7 | - | - | - | - |
| *Tubastraea* sp. | 7 | －20.3 (0.4) | ＋8.8 (0.2) | NE60 | －20.2 | ＋8.7 | - | 53 | 47 | - |
|  |  |  |  | NE70 | －19.9 | ＋9.2 | - | 50 | 50 | - |
|  |  |  |  | NE70 | －20.5 | ＋9.1 | - | 54 | 46 | - |
|  |  |  |  | NE80 | －20.0 | ＋9.0 | - | 50 | 50 | - |
|  |  |  |  | NE80 | －20.0 | ＋8.6 | - | 53 | 47 | - |
|  |  |  |  | SE40 | －21.1 | ＋8.8 | - | 60 | 40 | - |
|  |  |  |  | NW10 | －20.5 | ＋8.5 | - | 56 | 44 | - |
| *Anthopleura* sp. | 51 | －19.9 (0.4) | ＋9.2 (0.3) | NE0 | －20.3 | ＋9.4 | - | 53 | 48 | - |
|  |  |  |  | NE0 | －19.8 | ＋9.6 | - | 50 | 50 | - |
|  |  |  |  | NE0 | －20.6 | ＋9.3 | - | 55 | 45 | - |
|  |  |  |  | NE10 | －19.8 | ＋9.4 | - | 49 | 51 | - |
|  |  |  |  | NE20 | －20.2 | ＋9.1 | - | 52 | 48 | - |
|  |  |  |  | NE20 | －19.8 | ＋9.2 | - | 49 | 51 | - |
|  |  |  |  | NE20 | －20.3 | ＋9.3 | - | 53 | 47 | - |
|  |  |  |  | NE30 | －20.2 | ＋9.3 | - | 52 | 48 | - |
|  |  |  |  | NE30 | －20.1 | ＋9.1 | - | 51 | 49 | - |
|  |  |  |  | NE40 | －20.2 | ＋8.9 | - | 52 | 48 | - |
|  |  |  |  | NE40 | －20.4 | ＋9.4 | - | 53 | 47 | - |
|  |  |  |  | NE40 | －19.2 | ＋7.9 | - | 54 | 46 | - |
|  |  |  |  | NE60 | －18.7 | ＋9.4 | - | 42 | 58 | - |
|  |  |  |  | NE70 | －19.9 | ＋8.6 | - | 51 | 49 | - |
|  |  |  |  | NE70 | －20.2 | ＋9.0 | - | 52 | 48 | - |
|  |  |  |  | NE80 | －19.8 | ＋8.9 | - | 49 | 51 | - |
|  |  |  |  | NE80 | －20.0 | ＋8.9 | - | 51 | 49 | - |
|  |  |  |  | NE90 | －20.0 | ＋9.0 | - | 50 | 50 | - |
|  |  |  |  | NE90 | －19.0 | ＋9.0 | - | 44 | 56 | - |
|  |  |  |  | SW0 | －19.7 | ＋9.3 | - | 49 | 52 | - |
|  |  |  |  | SW0 | －20.0 | ＋9.2 | - | 50 | 50 | - |
|  |  |  |  | SW0 | －19.8 | ＋9.4 | - | 50 | 50 | - |
|  |  |  |  | SW10 | －19.9 | ＋9.2 | - | 50 | 50 | - |
|  |  |  |  | SW10 | －19.4 | ＋9.6 | - | 47 | 53 | - |
|  |  |  |  | SW10 | －19.7 | ＋9.4 | - | 49 | 51 | - |
|  |  |  |  | SW20 | －20.4 | ＋8.8 | - | 54 | 46 | - |
|  |  |  |  | SW20 | －20.3 | ＋9.3 | - | 53 | 47 | - |
|  |  |  |  | SW30 | －19.7 | ＋9.3 | - | 49 | 52 | - |
|  |  |  |  | SW30 | －20.2 | ＋9.1 | - | 52 | 48 | - |
|  |  |  |  | SW40 | －19.7 | ＋9.4 | - | 49 | 51 | - |
|  |  |  |  | SW40 | －19.7 | ＋8.9 | - | 49 | 51 | - |
|  |  |  |  | SW70 | －19.4 | ＋9.2 | - | 46 | 54 | - |
|  |  |  |  | SW80 | －19.9 | ＋9.2 | - | 49 | 51 | - |
|  |  |  |  | SW90 | －19.7 | ＋9.3 | - | 49 | 51 | - |
|  |  |  |  | SW90 | －19.8 | ＋9.0 | - | 49 | 51 | - |
|  |  |  |  | SE0 | －19.9 | ＋9.4 | - | 50 | 50 | - |
|  |  |  |  | SE10 | －19.9 | ＋9.1 | - | 50 | 50 | - |
|  |  |  |  | SE20 | －19.9 | ＋9.2 | - | 49 | 51 | - |
|  |  |  |  | SE30 | －20.3 | ＋9.0 | - | 53 | 48 | - |
|  |  |  |  | SE40 | －20.1 | ＋9.0 | - | 51 | 49 | - |
|  |  |  |  | SE50 | －19.8 | ＋9.3 | - | 49 | 51 | - |
|  |  |  |  | SE50 | －19.5 | ＋9.2 | - | 48 | 52 | - |
|  |  |  |  | SE60 | －20.2 | ＋9.2 | - | 52 | 48 | - |
|  |  |  |  | SE60 | －20.0 | ＋9.3 | - | 51 | 49 | - |
|  |  |  |  | SE70 | －20.3 | ＋9.1 | - | 53 | 48 | - |
|  |  |  |  | SE90 | －19.9 | ＋9.0 | - | 50 | 50 | - |
|  |  |  |  | SE100 | －19.7 | ＋9.4 | - | 49 | 51 | - |
|  |  |  |  | NW0 | －20.3 | ＋9.5 | - | 53 | 47 | - |
|  |  |  |  | NW10 | －20.5 | ＋9.2 | - | 54 | 46 | - |
|  |  |  |  | NW30 | －19.7 | ＋9.5 | - | 49 | 51 | - |
|  |  |  |  | NW40 | －20.7 | ＋9.6 | - | 56 | 44 | - |
| *Xenograpsus testudinatus* | 46 | －17.2 (1.0) | ＋8.2 (1.7) | NE0 | －17.3 | ＋8.3 | 21 | 25 | 54 | - |
|  |  |  |  | NE0 | －17.3 | ＋8.7 | 15 | 26 | 59 | - |
|  |  |  |  | NE0 | －17.9 | ＋9.1 | 11 | 34 | 55 | - |
|  |  |  |  | NE10 | －15.6 | ＋3.9 | 72 | 11 | 17 | - |
|  |  |  |  | NE20 | －17.7 | ＋9.2 | 10 | 32 | 58 | - |
|  |  |  |  | NE20 | －17.7 | ＋9.1 | 11 | 32 | 58 | - |
|  |  |  |  | NE20 | －17.7 | ＋8.4 | 19 | 30 | 51 | - |
|  |  |  |  | NE30 | －17.0 | ＋7.9 | 29 | 21 | 49 | - |
|  |  |  |  | NE30 | －17.6 | ＋9.0 | 12 | 30 | 58 | - |
|  |  |  |  | NE50 | －17.9 | ＋9.3 | 9 | 35 | 56 | - |
|  |  |  |  | SW0 | －18.6 | ＋8.6 | 15 | 38 | 48 | - |
|  |  |  |  | SW0 | －14.2 | ＋2.6 | 84 | 7 | 9 | - |
|  |  |  |  | SW0 | －18.1 | ＋9.0 | 11 | 36 | 53 | - |
|  |  |  |  | SW0 | －13.9 | ＋1.1 | 87 | 6 | 7 | - |
|  |  |  |  | SW10 | －17.9 | ＋8.6 | 16 | 33 | 52 | - |
|  |  |  |  | SW20 | －18.0 | ＋9.0 | 11 | 35 | 54 | - |
|  |  |  |  | SW30 | －18.3 | ＋9.0 | 11 | 37 | 52 | - |
|  |  |  |  | SW70 | －18.0 | ＋9.8 | 7 | 38 | 56 | - |
|  |  |  |  | SW70 | －17.4 | ＋9.1 | 12 | 27 | 61 | - |
|  |  |  |  | SW70 | －17.9 | ＋9.3 | 9 | 34 | 57 | - |
|  |  |  |  | SW80 | －16.9 | ＋8.0 | 29 | 20 | 51 | - |
|  |  |  |  | SW80 | －17.8 | ＋9.6 | 8 | 35 | 58 | - |
|  |  |  |  | SW90 | －16.7 | ＋8.2 | 26 | 18 | 56 | - |
|  |  |  |  | SW90 | －17.2 | ＋7.7 | 31 | 23 | 46 | - |
|  |  |  |  | SW90 | －18.3 | ＋9.8 | 6 | 42 | 52 | - |
|  |  |  |  | SW100 | －16.5 | ＋8.1 | 30 | 16 | 54 | - |
|  |  |  |  | SW100 | －18.8 | ＋8.9 | 12 | 40 | 48 | - |
|  |  |  |  | SE0 | －17.4 | ＋8.5 | 17 | 26 | 56 | - |
|  |  |  |  | SE0 | －16.2 | ＋5.7 | 57 | 15 | 28 | - |
|  |  |  |  | SE0 | －17.3 | ＋8.6 | 17 | 25 | 58 | - |
|  |  |  |  | SE10 | －17.9 | ＋8.9 | 12 | 33 | 55 | - |
|  |  |  |  | SE10 | －17.8 | ＋9.7 | 7 | 36 | 57 | - |
|  |  |  |  | SE10 | －16.3 | ＋7.1 | 47 | 16 | 37 | - |
|  |  |  |  | SE20 | －17.2 | ＋8.5 | 18 | 24 | 57 | - |
|  |  |  |  | SE30 | －17.7 | ＋8.8 | 14 | 31 | 55 | - |
|  |  |  |  | SE70 | －16.5 | ＋7.7 | 37 | 17 | 46 | - |
|  |  |  |  | SE70 | －17.4 | ＋8.7 | 16 | 26 | 58 | - |
|  |  |  |  | SE70 | －16.7 | ＋8.5 | 20 | 18 | 62 | - |
|  |  |  |  | SE70 | －16.8 | ＋7.6 | 35 | 19 | 46 | - |
|  |  |  |  | SE80 | －16.6 | ＋7.5 | 39 | 18 | 43 | - |
|  |  |  |  | SE80 | －17.1 | ＋8.4 | 21 | 23 | 56 | - |
|  |  |  |  | SE90 | －16.6 | ＋7.5 | 38 | 18 | 44 | - |
|  |  |  |  | SE90 | －16.1 | ＋6.9 | 51 | 15 | 33 | - |
|  |  |  |  | NW20 | －17.4 | ＋8.4 | 19 | 26 | 55 | - |
|  |  |  |  | NW30 | －17.0 | ＋9.5 | 9 | 23 | 68 | - |
|  |  |  |  | NW40 | －17.8 | ＋9.4 | 8 | 34 | 58 | - |
| *Anachis misera* | 13 | －18.0 (0.6) | ＋8.8 (0.3) | NE0 | －16.8 | ＋9.5 | - | 19 | 36 | 45 |
|  |  |  |  | SW20 | －18.0 | ＋8.8 | - | 28 | 42 | 30 |
|  |  |  |  | SW20 | －18.1 | ＋8.8 | - | 29 | 42 | 30 |
|  |  |  |  | SW30 | －18.3 | ＋8.8 | - | 30 | 42 | 28 |
|  |  |  |  | SW100 | －16.6 | ＋9.0 | - | 18 | 35 | 47 |
|  |  |  |  | SE10 | －17.9 | ＋8.5 | - | 27 | 40 | 33 |
|  |  |  |  | SE20 | －17.9 | ＋8.7 | - | 27 | 41 | 32 |
|  |  |  |  | SE30 | －18.3 | ＋8.7 | - | 30 | 41 | 29 |
|  |  |  |  | SE40 | －18.9 | ＋8.3 | - | 32 | 38 | 30 |
|  |  |  |  | SE60 | －18.0 | ＋8.7 | - | 27 | 41 | 31 |
|  |  |  |  | NW10 | －18.8 | ＋8.7 | - | 33 | 41 | 27 |
|  |  |  |  | NW30 | －17.9 | ＋9.1 | - | 28 | 43 | 30 |
|  |  |  |  | NW40 | －18.0 | ＋9.1 | - | 28 | 43 | 29 |
| *Ergalatax contratus* | 12 | －17.1 (0.4) | ＋8.7 (0.7) | SW20 | －17.2 | ＋9.1 | - | 22 | 40 | 39 |
|  |  |  |  | SW30 | －17.2 | ＋9.4 | - | 22 | 39 | 39 |
|  |  |  |  | SW40 | －17.1 | ＋8.1 | - | 21 | 33 | 46 |
|  |  |  |  | SW90 | －17.2 | ＋8.4 | - | 21 | 36 | 43 |
|  |  |  |  | SW90 | －17.0 | ＋9.4 | - | 21 | 38 | 41 |
|  |  |  |  | SW100 | －16.3 | ＋8.9 | - | 16 | 30 | 54 |
|  |  |  |  | SW100 | －17.4 | ＋8.1 | - | 22 | 35 | 43 |
|  |  |  |  | SW100 | －16.7 | ＋8.5 | - | 19 | 33 | 49 |
|  |  |  |  | SE50 | －16.7 | ＋9.6 | - | 18 | 34 | 48 |
|  |  |  |  | SE70 | －17.1 | ＋7.6 | - | 20 | 30 | 50 |
|  |  |  |  | SE70 | －17.3 | ＋7.8 | - | 21 | 32 | 47 |
|  |  |  |  | SE70 | －17.7 | ＋9.1 | - | 26 | 42 | 32 |

^1^N is the sample number of isotopic measurement.
